# Risk Communication in the COVID-19 Outbreak: Two Sides of the Same Coin

**DOI:** 10.1017/dmp.2020.327

**Published:** 2020-09-09

**Authors:** Hamid Safarpour, Iman Farahi-Ashtiani, Davoud Pirani, Bayram Nejati, Meysam Safi-Keykaleh

**Affiliations:** Department of Nursing, Faculty of Nursing and Midwifery, Ilam University of Medical Sciences, Ilam, Iran; Department of Health in Disasters and Emergencies, School of Public Health and Safety, Shahid Beheshti University of Medical Sciences, Tehran, Iran; Malayer School of Nursing, Hamadan University of Medical Sciences, Hamadan, Iran

**Keywords:** COVID-19, disasters, epidemics, infectious disease risk communication

The appearance of a new infectious disease with an epidemic potential usually ignites the society, media, academic centers, and political circles.^1^ Currently, the coronavirus disease (COVID-19) pandemic is at the focus of attention of world health systems, media, policy-makers, and academic centers. Despite widespread prevention measures, the whole world is anxious about the destructive outcome of the COVID-19 pandemic.

One of the most significant and effective interventions in the response of public health to any event is establishing an active engagement with what is known, as well as what it takes to get more information, in order to minimize the negative consequences. The outbreak of SARS-CoV-2 has challenged public health systems and their capacity for establishing effective engagement with the communities.^2^ Production, transmission, and spread of the information related to COVID-19 are accompanied by remarkable rumors and misinformation.^3^ Misinformation disrupts people’s perception of risk and diverts community from understanding the original risk.^4^ Meanwhile, ineffective communication in risk and unreliability situations could lead to consequences, such as the loss of trust and reputation, socioeconomic effects, psychological effects, and, in worst case scenarios, loss of lives.^2^

The production and release process of scientific and public health information and the adoption of this information by the media as the main source of correct information for the citizens include various stages, each of which could cause exaggeration or misinformation.^1,5^ The rapid increase of Internet-based health news and information might encourage the selection of media and academic research articles that exaggerate the power of causal inference.^1^ Reporting the state of affairs to the media in due time and in an honest way by the authorities can lead to dissemination of correct and timely information. Trustworthiness of official information channels about COVID-19 may be influenced by the communities’ trust on those official channels. It is recommended that communication behaviors be improved to solve miscommunication at times of pandemics like SARS-CoV-2. Because of the constant changes in the effects of COVID-19 and the exaggerated public perception of its mortality risk because of the difficulty of estimating the mortality rate,^6^ the response of global media to COVID-19 remains unbalanced.^1^ To protect global health security, it is essential that developed dialogue and altruistic intentions with appropriate authorities are focused by media.^1^

Effective communication not only brings about a decrease in the risk of misbehavior, such as unnecessary hospital visits, but it also assists in removing fake information and discrimination against the patients and their visitors.^5^ Globally, the total number of social media users is estimated to grow to 3.29 billion users in 2022, which will be 42.3% of the world’s population.^7^ Moreover, data gathered on the global television market showed that there were 1.67 billion pay TV households worldwide in 2018. Therefore, 1 way to ensure proper communication is to use social media, radio, and TV channels and ensure the constant presence of the media using the World Health Organization (WHO) principles for effective communication: accessible, actionable, credible and trusted, relevant, timely, and understandable.^8^

Risk communication and community engagement contribute to the prevention of infodemics (too much information about a problem, which makes it difficult to identify the solution)^2^ and increase the chance of health advice following for self-protection by creating trust in response.^2,9,10^ Academic circles strongly advise to avoid panic^6^ since fake news lead to undue fear or lack of enough sensitivity in taking preventive measures, thus increasing spread of the disease.^5^ Establishing honest engagement with the people through licensed official media with cooperation of the Ministry of Health and other governmental agencies could prevent miscommunication and misunderstanding.^11^ People have every right to receive sufficient information about the risks that threaten their health to be able to conceive of the situation. Risk perception among the affected population is usually different from that of the experts and officials. Risk communication and community engagement for monitoring, reporting the cases, tracing the contacts, taking care of patients, offering clinical service, and compiling local support for any kind of logistical and operational demand are necessary for responding. Based on the interim guidance of the WHO, all governments can engage their communities in many ways, including stabilizing communication proactively, identifying community influencers (community and religious leaders, health workers, and traditional healers), and anticipating peoples’ needs, concerns, beliefs, and attitudes.^2^ Risk communication and effective community engagement can minimize social disorder, as well as protect health, the economy, and tourism.^2^

Disaster risk communication, in general, and the case of COVID-19, in particular, resemble 2 sides of a coin. On 1 side, there is timely, correct, and trusty risk communication, which contributes to a timely and proper response by public participation. On the other side, there is flawed risk communication and faulty communications, which result in an ineffective response, making the situation worse. Therefore, it is recommended that the health systems of countries provide correct, timely, and trusted information and training packages for their people through media with the participation of the WHO and taking into account the cultural contexts, disease and epidemic status, and population features, including various age groups as well as education and awareness levels. Furthermore, it is recommended that the government officials encourage the people to participate in the prevention and care processes via social media and other media channels.
